# ID16B: a hard X-ray nanoprobe beamline at the ESRF for nano-analysis

**DOI:** 10.1107/S1600577515019839

**Published:** 2016-01-01

**Authors:** Gema Martínez-Criado, Julie Villanova, Rémi Tucoulou, Damien Salomon, Jussi-Petteri Suuronen, Sylvain Labouré, Cyril Guilloud, Valentin Valls, Raymond Barrett, Eric Gagliardini, Yves Dabin, Robert Baker, Sylvain Bohic, Cédric Cohen, John Morse

**Affiliations:** a European Synchrotron Radiation Facility, 71 Avenue des Martyrs, 38000 Grenoble, France

**Keywords:** X-ray nanoprobe, X-ray fluorescence, microspectroscopy

## Abstract

Within the framework of the ESRF Phase I Upgrade Programme, a new state-of-the-art synchrotron beamline ID16B has been recently developed for hard X-ray nano-analysis. The construction of ID16B was driven by research areas with major scientific and societal impact such as nanotechnology, earth and environmental sciences, and bio-medical research. Based on a canted undulator source, this long beamline provides hard X-ray nanobeams optimized mainly for spectroscopic applications, including the combination of X-ray fluorescence, X-ray diffraction, X-ray excited optical luminescence, X-ray absorption spectroscopy and 2D/3D X-ray imaging techniques. Its end-station re-uses part of the apparatus of the earlier ID22 beamline, while improving and enlarging the spectroscopic capabilities: for example, the experimental arrangement offers improved lateral spatial resolution (∼50 nm), a larger and more flexible capability for *in situ* experiments, and monochromatic nanobeams tunable over a wider energy range which now includes the hard X-ray regime (5–70 keV). This paper describes the characteristics of this new facility, short-term technical developments and the first scientific results.

## Abstract 2

ID16B is a versatile hard X-ray nanoprobe devoted to X-ray nano-analysis. It combines X-ray fluorescence, X-ray diffraction, X-ray absorption spectroscopy and 2D/3D X-ray imaging techniques.

## 1. Introduction   

**Figure 1:**
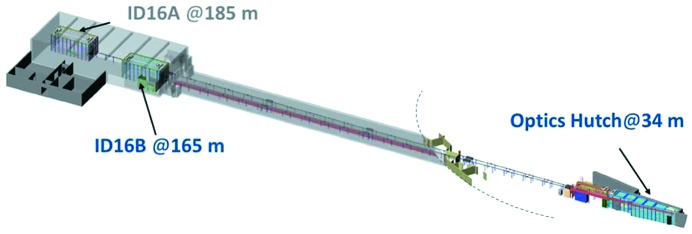
Schematics of the general layout of the UPBL4 NINA.

New synchrotron nanoprobes are becoming available with smaller beam-spot sizes, down to a few tens of nanometers (Chen *et al.*, 2014 ▸; Winarski *et al.*, 2012 ▸; Maser *et al.*, 2014 ▸; de Jonge *et al.*, 2014 ▸; Johansson *et al.*, 2013 ▸; Somogyi *et al.*, 2013 ▸): this is driven by the large demand for higher spatial resolution from many key scientific areas such as nanotechnology, earth, environmental and life science, and materials science (Bohic *et al.*, 2012 ▸; Martínez-Criado *et al.*, 2014 ▸; Trushin *et al.*, 2010 ▸; Segura-Ruiz *et al.*, 2014 ▸; Somogyi *et al.*, 2015 ▸; Winarski *et al.*, 2012 ▸; Schroer *et al.*, 2010 ▸). Within the framework of the Phase I Upgrade Programme of the ESRF (http://www.esrf.eu/about/upgrade), among the eight completely new beamlines developed, the UPBL4 NINA project comprises two independent long nanoprobes which operate in parallel: ID16A for Nano-Imaging, located at 185 m from the source, and ID16B for Nano-Analysis, at 165 m. Fig. 1 ▸ shows the general layout: the satellite building that houses both end-stations, and the common optics hutch which accommodates independently the optical elements for each beamline with an intricate design. Both beamlines exploit the very low vertical emittance of the ESRF source, using the same basic principle for the horizontal focusing, *i.e.* a high-β straight section coupled with a horizontal secondary source, offering flexibility in terms of spatial resolution and photon flux. Whereas ID16A mainly addresses problems in biology, biomedicine and nanotechnology using fluorescence analysis and nano-tomography with ultimate hard X-ray focusing optics (http://www.esrf.eu/about/upgrade), in a complementary fashion ID16B offers a multi-modal approach for the full and simultaneous characterization at the nanoscale of diluted heterogeneous samples with non-destructive investigation of the spatial distribution, concentration and speciation of trace elements correlated to their morphology and crystallographic orientations at the nano­meter level.

From a historical viewpoint, ID16B merges the natural evolution of the earlier ID22 EH1 station and the experience of a nano-imaging pilot project operated on ID22 EH2 (Martínez-Criado *et al.*, 2014 ▸; Tucoulou *et al.*, 2008 ▸). ID16B was opened to users in April 2014, and its multimodal scheme provides complementary sets of information using the following X-ray methods:

(i) X-ray fluorescence (XRF).

(ii) X-ray absorption spectroscopy (XAS).

(iii) X-ray diffraction (XRD).

(iv) X-ray excited optical luminescence (XEOL).

(v) Magnified phase contrast imaging.

(vi) X-ray beam-induced current (XBIC) mapping.

(vii) Extension of the above methods to three dimensions (generalized computed tomography).

In addition to these approaches, the high penetration power of the ID16B X-ray beam enables the integration and development of controlled sample environments. Examples include a micro-heater (up to 1200°C) for tomography acquisitions, a He cryostat (−263°C) for XEOL and XRF measurements and a Linkam stage (variable to −193°C) for freezing applications (Martínez-Criado *et al.*, 2007 ▸; http://www.linkam.co.uk).

ID16B provides a highly focused beam (50–100 nm) and with a large (>30 mm) working distance to the last Kirk­patrick–Baez (KB) providing enough space for a sample environment. In the next section the major technical aspects are summarized.

## 2. Instrumentation   

### 2.1. X-ray source   

Undulator sources are located in the high-β 6 m straight section of the ID16 port; these are canted symmetrically at angles of ±2.7 mrad. A revolver undulator system is located downstream for ID16A, while ID16B has a fully tunable U26-type in-vacuum undulator of 2.5 m length, 26 mm period and 6.5 mm minimum gap. Table 1 ▸ lists the most relevant undulator parameters. The fundamental energy is indicative only as it neglects the finite beam size and emittance.

**Figure 2:**
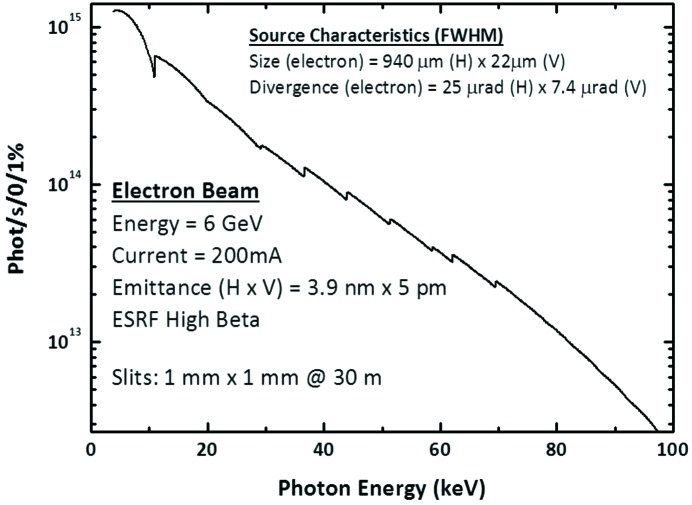
Output spectrum of the U26 in-vacuum undulator shown as photons s^−1^ (0.1% bandwidth)^−1^ (equivalent to the position and normal slit gaps of the primary slits) from the center of the undulator.

The photon flux emitted by the undulator, calculated at 30 m from the source through a 1 mm × 1 mm pinhole, is presented in Fig. 2 ▸. The ESRF storage ring electron beam has an energy of 6.037 GeV, with a relative energy spread of 0.001, and maximum current of 200 mA. The vertical (horizontal) emittance, β values and dispersion are 5 pm (3.9 nm), 3 m (37.2 m) and 0 (0.03 m), respectively.

### 2.2. Beamline optical layout   

**Figure 3:**
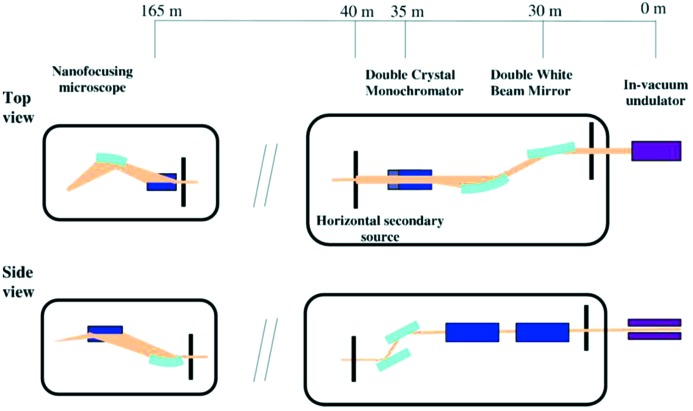
General optical layout of ID16B with the primary optical elements.

Fig. 3 ▸ shows the layout of ID16B and the location of the main optical elements. While the primary beamline optics [double white beam mirror (DWM) and double-crystal monochromator (DCM)] are placed as close to the source as possible (∼30 m and 35 m, respectively) to preserve the coherence of the beam and to minimize beam instabilities, the KB mirror nanofocusing optics at 165 m are located very close to the sample (∼0.1 m) to obtain a high degree of demagnification. In order to collect the large angular cone, the primary optical elements that generally introduce wavefront distortions by imperfections need to be installed as close as possible to the source. In the same way, putting the primary optics close to the source results in a reduction of microradian angular fluctuations, which induce movements of the effective point source, causing deviations of the focused beam. For the horizontal focusing geometry, on the other hand, two alternative solutions are possible: an intermediate focus (by combining a horizontally focusing mirror with a precision horizontal slit at 40 m) or an aperture to delimit the effective source size (a precision horizontal slit at 40 m). The fixed-exit horizontal DWM (Si substrate with Pd and Pt coatings, 6 mm offset and 2.6 mrad nominal angle of incidence) is typically used for harmonic rejection with a dynamically bent second mirror to create the horizontal secondary source in combination with the precision slit located at 40 m. The fixed-exit vertical DCM (with −12.5 mm offset) is essential for spectroscopy and diffraction experiments, which require an extremely high mechanical stability and a fixed-exit capability to keep the nanobeam stable during an entire XAS scan (ideally the parallelism between the monochromator crystals should be better than 0.05 µrad over a 1 keV scan). Initially ID16B re-used the DCM from the earlier ID22 (Martínez-Criado *et al.*, 2012 ▸) beamline, but following a new development the DCM in use today on ID16B is equipped with two sets of crystals: Si (111) for the 4–37 keV and Si (311) for the 7–72 keV energy ranges. The errors of parallelism between the crystals reach a few µrad per keV, but to a large extent these errors are reproducible.

After the DCM two safety apertures are used to protect the downstream beam vacuum transfer line that carries the beam to the experimental hutch located in the satellite building. In addition, extractable beam position monitors are installed downstream of each optical element: five off-line beam viewers for beam diagnostics purposes, and two X-ray beam position monitors for on-line monitoring dedicated to beam steering and/or corrections.

In general, three different beam operation modes are distinguished from the primary elements situated in the optics hutch: (i) low-energy monochromatic mode (Δ*E*/*E* ≃ 10^−4^) using both DWM and DCM; (ii) high-energy monochromatic mode (Δ*E*/*E* ≃ 10^−4^) with DCM; and (iii) pink beam mode (Δ*E*/*E* ≃ 10^−2^) with DWM.

### 2.3. End-station   

**Figure 4:**
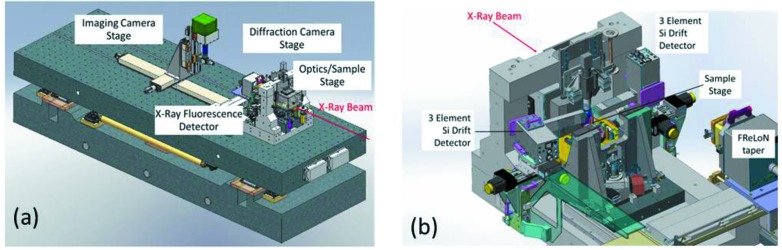
(*a*) Overview of the experimental table of ID16B end-station. (*b*) Amplified view of the nanoprobe. The X-ray beam is focused down to nanometer scale using KB optics located below the granite bridge structure.

Because the satellite building houses the end-station, special attention has been paid to its vibrations and thermal stability. The building is single storey with an aerodynamic design that prevents air-flow-related effects. It is built on a special concrete slab designed to nullify ‘curling’ from temperature gradient variations. All services and HVAC (heating, ventilating and air conditioning), mechanical vacuum pumps *etc*. that could induce vibrations are located far from the slab. Taking into account that thermal changes are the main source of long-term instabilities during experiments, a design with two successive layers was adopted: ±0.5°C for the satellite building and ±0.05°C for the experimental hutch. All sources of heat and/or vibrations (chillers, vacuum pumps, electronics *etc*.) are situated remotely from the end-station. An overview of the experimental arrangement of the nano­probe is depicted in Fig. 4 ▸. Compared with the previous nanoprobe setup of ID22NI, the actual ID16B end-station provides better performance in terms of lateral resolution (∼50 nm), energy range (5–70 keV), range of detection schemes, monochromatic operation for absorption measurements, and its *in situ* capabilities. As shown in Fig. 4(*a*) ▸, the apparatus is mounted on a common table, reducing possible vibrations with respect to each other of the various components. Superposed on a concrete base, the table consists of 4.4 m-long, 1.5 m-wide and 0.3 m-thick granite, which is motorized in the vertical direction to adapt the equipment height to the pink or monochromatic beam modes. Vibration measurements show only a weak and damped oscillation of the floor vibrations around 50 Hz. Using viscoelastic materials, passive damping systems like damping plates, pads and links, which are compatible with alignment operation purposes, are installed to attenuate and absorb vibrations.

**Figure 5:**
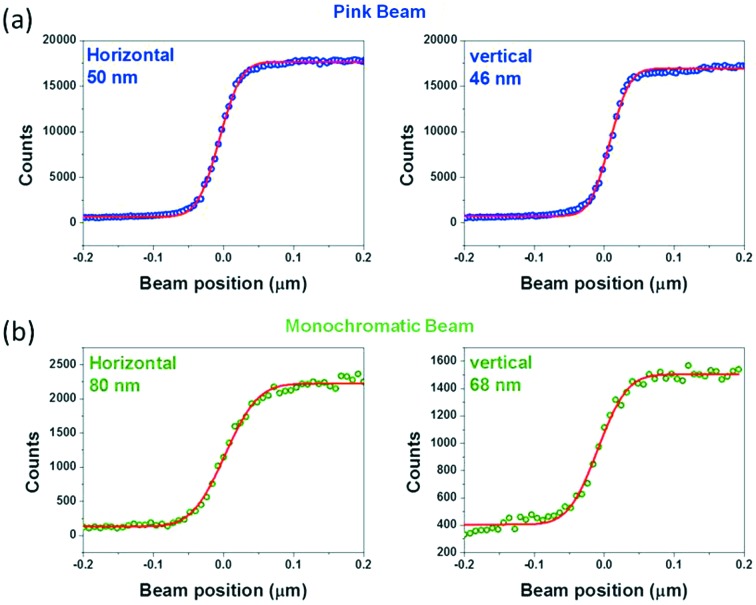
Focused beam integral profiles taken at 29.6 keV in pink beam (*a*) and 29.6 keV in mono beam mode (*b*) by means of Au knife-edge scans. Solid circles represent the raw data and solid lines represent the respective fits.

Fig. 4(*b*) ▸ shows the granite structure on which are supported the KB optics, sample stage, visible-light microscope and X-ray fluorescence detectors. In addition there are two stages for the scintillator screen cameras used for X-ray diffraction (FReLoN camera with fiber optic taper coupling to the scintillator) and X-ray imaging (FReLoN 4M or PCO edge 5.5 using lens coupling) (Labiche *et al.*, 2007 ▸). Efficient achromatic X-ray nanofocusing to ∼50 nm is obtained in a routine fashion using the elliptical-shaped multilayer-coated KB mirror system which was previously installed on ID22NI. Such dynamically bent KB mirrors can be operated in pink beam mode with Bragg diffraction (Δ*E*/*E* = 10^−2^) or in monochromatic beam mode under grazing incidence (Δ*E*/*E* = 10^−4^). A complete description of the nanofocusing optics can be found elsewhere (Tucoulou *et al.*, 2008 ▸; Barrett *et al.*, 2011 ▸). The resulting spot sizes at the focal plane for both configurations are shown in Fig. 5 ▸. The vertical mirror images the undulator source (of size ∼25 µm FWHM), whereas the secondary source (slit ≃ 50 µm FWHM) is used in the horizontal direction. At the time of writing a second KB system is in the design phase: this will be operated under external total reflection.

### 2.4. Sample stage   

**Figure 6:**
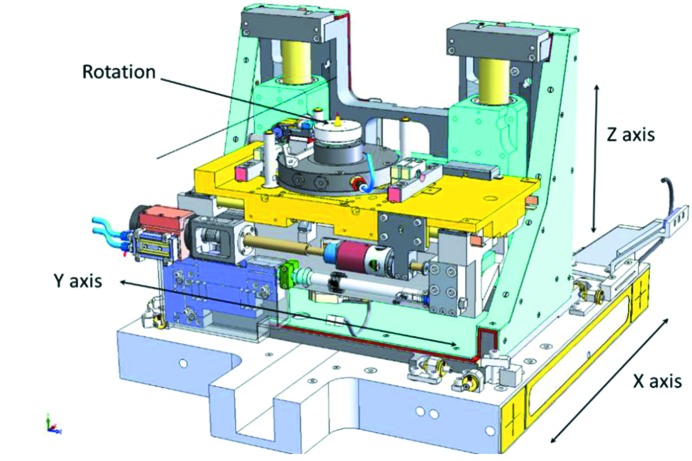
Movements of the sample stage: *X*, *Y* and *Z* translations and the nano-spindle rotation stage.

As shown in Fig. 6 ▸, the sample stage is a unique combination of three-axis, motorized translation stages integrated with an air-bearing nano-spindle rotation stage (PIC ISO 3R). The *X*-axis stage is a granite block equipped with air pads and translated *via* a linear motor (ETEL motion controller). It carries the vertical and horizontal scanning stages (*Y*, *Z*) as well as the PIC nano-spindle. This design ensures precise, smooth and continuous motions with submicrometer resolution. The stages provide 320 mm, 40 mm and 50 mm of *X*, *Y* and *Z* travel range, respectively. With a 30 kg load capacity, the horizontal and vertical stages have 25 and 37 nm resolution, respectively. In addition, the *Y*-axis stage incorporates a piezoelectric actuator (PI electronics) with 90 µm of fine travel. The rotation stage has ±1 µrad resolution with ∼25 nm bidirectional radial and axial errors.

**Figure 7:**
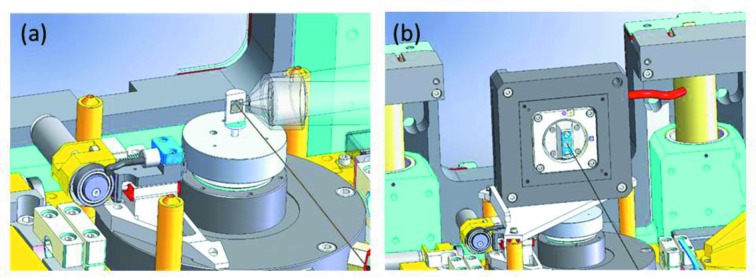
Sample supports for 3D (*a*) and 2D (*b*) schemes for XRF and XRD magnified imaging acquisitions, respectively.

The PIC air-bearing spindle is typically used for 3D acquisitions in XRF, XRD and magnified tomography (Fig. 7*a* ▸). The spindle is driven by a brushless DC motor and an ETEL controller. For this configuration, the X-ray fluorescence detectors are usually mounted at 90° with respect to the X-ray beam axis and the main *Y*–*Z* stages are used for scanning the specimen. For magnified tomography the sample is moved out of the focus along the direction of the X-ray beam using the *X* translation stage. Depending on the nature of the sample, various mechanical support options are possible. Huber specimen carriers for goniometer head series 1000 can be used for glass and quartz capillaries. PEEK frames to carry Si_3_N_4_ membranes or custom Al holders are available for flat specimens. Using a kinematic mount on top of the horizontal and vertical stages, a *Y*–*Z* piezo flexure nanopositioning stage (Physik Instruments) can be mounted on top of the sample stage (Fig. 7*b* ▸) for 2D XRF, XRD and magnified imaging. The nano-piezo features linear travel ranges of 100 µm × 100 µm with nanometer resolution. This configuration is adapted to specimens deposited on flat substrates (Si_3_N_4_ windows, electron microscopy grids *etc*.). The samples are easily mounted from the back of the PI stage using a magnetic kinematic support. Scanning is at normal incidence, *i.e.* the incident X-ray beam is approximately perpendicular to the sample surface. For this geometry the fluorescence detectors are positioned in the horizontal plane typically at 15° with respect to the sample surface. As part of the data collection scheme, a fast acquisition approach, the so-called continuous scanning mode, has been implemented that enables two-dimensional data to be recorded from multiple detectors on a millisecond timescale.

### 2.5. Detection schemes   

#### 2.5.1. X-ray fluorescence   

Depending on the detection energy range, several options are available for XRF. Silicon drift detectors (SDDs) are commonly used to quantitatively measure XRF signals from the samples of energies from 2 keV up to 25 keV at which energy the detector efficiency has fallen to ∼20% due to the limited silicon X-ray absorption; high-energy measurements are made in preference with a germanium diode-array spectroscopy detector. Both detector types are operated in association with the XIA-XMAP (PXI bus format) digital pulse processors (http://www.xia.com).

For energies below 25 keV the simplest configuration at ID16B comprises two single-element SII Nano Technology Vortex-90EX SDDs, each with an active silicon area of about 50 mm^2^ and 350 µm thickness, and with 25 µm-thick beryllium windows. These detectors feature excellent resolution (<140 eV FWHM at 5.9 keV) and high count rate capability: at a pulse processor peaking time of 0.25 µs an output count rate of 600 kcounts s^−1^ is achieved. In addition, it is possible to rapidly exchange both of these detectors with two identical detectors from SGX Sensortech, each comprising three-element SDD array modules of 450 µm silicon thickness and with 30 µm-thick beryllium windows. The active area of each SDD is 83 mm^2^, defined by a pure zirconium ‘on-chip’ collimator. The SDD array modules (produced by PNDetector) provide a resolution of 145 eV FWHM at 5.9 keV with a peak-to-background ratio of >3000:1 and an output count rate capability of over 400 kcounts s^−1^ per SDD. Since the experimental station is temperature stabilized at 23°C to within ±0.05°C to ensure dimensional stability of critical beamline optical components, to avoid local temperature variations no significant exchange of heat must occur either to or from the detector housing and its surrounding ambient environment. To ensure this, all power dissipated within the SGX Sensortech detector housing is removed using a recirculating fluid circuit to a chiller unit (operated at the stabilized ambient hutch temperature) which is placed outside of the experimental hutch.

Particular to our station is the possibility to operate up to ∼70 keV. At such high energies, only germanium crystal based detectors have sufficient X-ray stopping power and energy resolution for fluorescence spectroscopy. For this reason a detector comprising a close-packed array of germanium diodes is also available, and this detector can be rapidly exchanged to occupy the position of one of the two SDD detectors. The germanium detector consists of eight discrete circular Canberra ‘Ultra-LEGe’ diode crystals each of nominal 50 mm^2^ area, arranged in double line, vertical array geometry. The crystals are 5 mm thick with a single 25 µm beryllium window. The detector offers excellent performance: 140 (550) eV FWHM at 5.9 (122) keV, a peak-to-background higher than 1000:1, and a throughput count rate capability >10^5^ counts s^−1^ channel^−1^ for short peaking times (<1 µs). A unique feature of this detector is that its cryogenic cooling (∼80 K) is achieved using an integrated pulse-tube cooler: this is the first X-ray multielement detector commercially supplied using this technique. This cooling method avoids perturbations to the precisely stabilized temperature at the sample stage which would arise from thermal losses associated with the use of a liquid-nitrogen-cooled system: as for the SDD array detectors, the power dissipated within the Canberra detector housing (∼100 W) is transferred outside the experimental hutch by a recirculating fluid circuit.

#### 2.5.2. X-ray diffraction   

As previously used on ID22 (Martínez-Criado *et al.*, 2012 ▸), the fiber-optic taper version of the FReLoN F_4320T camera is installed in transmission geometry for XRD acquisitions (Labiche *et al.*, 2007 ▸). A 50 µm-thick gadolinium oxysulphide powder scintillator screen converts the X-rays into visible-light photons, which are guided by a tapered fiber-optic bundle that is hard-bonded to a Kodak CCD sensor. The 3.3:1 demagnification provided by the tapered fiber optic results in an effective pixel size of 51 µm square and a total 109 mm^2^ field of view. The camera has a response of 3.9 ADU per incident 20 keV X-ray photon and a peak detective quantum efficiency of 0.6 at 20 keV: details of this camera system are given by Labiche *et al.* (2007 ▸). A photodiode is integrated within the beamstop support to measure the transmitted beam intensity simultaneously with the camera exposures.

#### 2.5.3. Magnified imaging   

Two detection systems are available to meet the demands of the user applications: *in situ* conditions, high or low photon flux experiments. Both systems share a common design: a scintillator screen which converts X-rays into a visible-waveband image that is magnified by optics [usually from Optique Peter (http://www.optiquepeter.com)] onto a CCD- or CMOS-sensor camera.

The FReLoN 4M F_4320 camera system that was commonly used in magnified tomography on the hard X-ray nanoprobe of ID22 (Martínez-Criado *et al.*, 2012 ▸) uses a ×3.1 magnification eyepiece giving an effective pixel size of 7.6 µm square with a field of view of 2048 × 2048 pixels. For fast image acquisition on ID16B, a PCO Edge 5.5 CMOS camera has been recently installed. It presents excellent characteristics of frame readout speed (100 frames s^−1^); spatial resolution (H × V) of 2560 × 2160 pixels with 6.5 µm × 6.5 µm pixel size; quantum efficiency > 60%; and a dynamic range of 27000:1. This detection system is important for medium-speed high-quality scans that cannot be achieved with the slower readout FReLoN camera. In both cases the high-resolution X-ray imaging microscope includes a motorized, triple scintillator mount; motorized triple objective lenses (4×/0.16–10×/0.30–10×/0.40); and motorized camera rotation to ensure perfect angular alignment of the CCD or CMOS pixel array with the sample scan axes.

## 3. Examples of scientific applications   

### 3.1. Fate of Ag nanoparticles on sunflower roots   

Silver nanoparticles (AgNPs) are currently the most widely commercialized nanomaterial. As a result, the increasing likelihood of accidental or incidental release of AgNPs into the environment requires a thorough understanding of the long-term impact of their interaction with plant life. The high specific surface area and reactivity of these nanoparticles has raised concerns about their evolution, transport and toxicity in the environment. In particular, as they can enter into the food chain, it is very important to trace AgNPs taken up from the soil by plants and to analyse the chemical states of the metallic elements.

In a few recent studies the potential phytotoxicity of AgNPs has been investigated, and negative effects were reported after short-term exposure. For example, citrate-coated AgNPs inhibited *Arabidopsis thaliana* seedling root elongation with a linear dose response after two week exposure, although seed germination was not affected (Geisler-Lee *et al.*, 2012 ▸). Similar effects were observed for *Lolium multiflorum*, with inhibition of plant growth on agar medium becoming more pronounced as the concentration of AgNPs increased (Yin *et al.*, 2011 ▸). As an alternative to root uptake, penetration through stomata in lettuce plant leaves has also demonstrated foliar uptake of AgNPs from an atmospheric source (Larue *et al.*, 2014 ▸). Plants in the environment are likely to be exposed to much lower AgNP concentrations, but the effect of AgNPs at such low concentrations or after long-term exposure has received only limited attention. Furthermore, whereas uptake studies have been conducted with several types of nanoparticles, the internalization and speciation in plants remain poorly understood.

**Figure 8:**
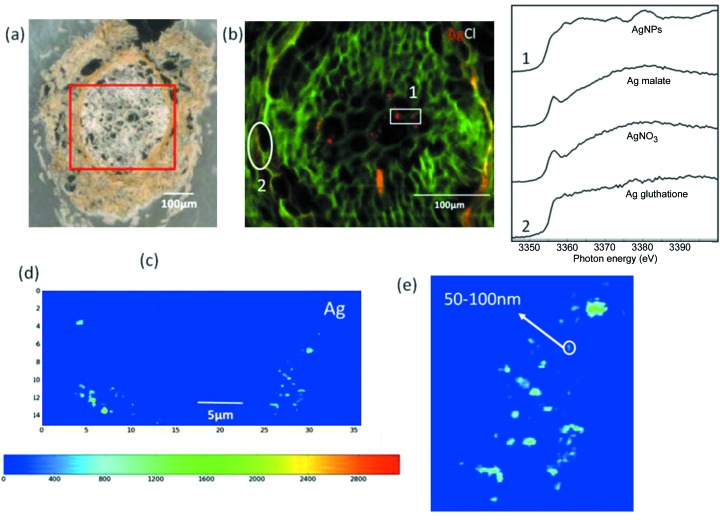
(*a*) Optical image of a cut sunflower root. (*b*) False color element maps of Cl and Ag in the sunflower root. (*c*) XANES data on AgNPs in the vascular region taken at ID21. (*d*) False colour element maps of Ag taken at ID16B in the region 1 of (*b*). (*e*) Zoom on the right-hand part of the map shown in (*d*).

At ID16B the fate of AgNPs on sunflower roots has been studied using nano-XRF imaging. Sunflower plantlets were exposed to AgNPs for three days. The distribution and speciation of Ag was investigated first at beamline ID21 using a submicrometer (800 nm) beam at 3.56 keV incident energy (corresponding to the Ag *L*_III_-edge). Figs. 8(*a*)–8(*c*) ▸ show an optical image of the sample, element distribution maps and XANES data.

The results from the ID21 measurements show that there is elemental silver in the plant vascular region [Figs. 8(*b*) and 8(*c*) ▸] suggesting this might be NPs or NPs aggregates. To verify this, element maps with higher lateral resolution (50 nm beam size) at 29.6 keV incident energy, *i.e.* above the Ag *K*-edge, were collected on ID16B. The distribution of Ag shown in Figs. 8(*d*) and 8(*e*) ▸ clearly reveals the presence of Ag single NPs and aggregates of Ag NPs having different sizes (50 nm < aggregates < 500 nm). This is the first time that the localization of AgNPs in the vascular region of plants has been studied using nano-XRF. Future ID16B technical developments in spectroscopy will enable XANES measurements on NPs to elucidate chemical modifications at the nanoscale.

### 3.2. Submicrometer heterogeneities in diamond inclusions   

During the growth of diamonds, different minerals can be trapped in inclusions preserving the original composition of the deep Earth. Consequently they are unique direct sources of information about the chemical and physical conditions within the transition zone and lower mantle. There are only a few sources of ultra-deep diamonds in the world: one of these is located in the Juina region, in the northwest of the state of Mato Grosso in Brazil, near the south-western edge of the Amazonian craton.

Nano-XRF studies of inclusions in a natural diamond from the Juina region were recently carried out at ID16B. The experiment probed regions where Raman scattering measurements showed the presence of ringwoodite. Typically produced in the laboratory, this Fe-enriched phase has been recently discovered for the first time in natural terrestrial materials (Pearson *et al.*, 2014 ▸), suggesting the potential presence of large amounts of hydrous phases, which could have a key role in plate tectonics and terrestrial magnetism. Extending the investigation to other diamonds containing ringwoodite inclusions might provide further confirmation of this observation, adding relevant information about the deep Earth.

**Figure 9:**
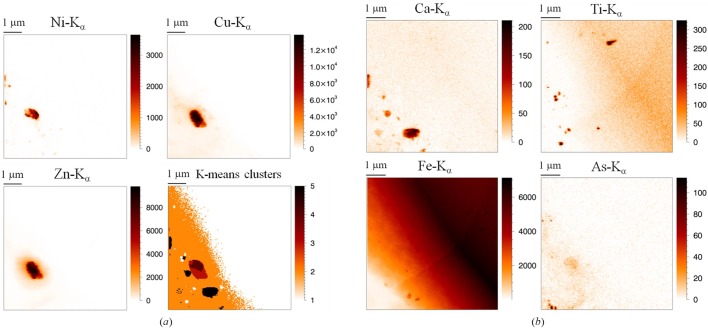
(*a*) XRF maps of Ni, Cu and Zn, the Cu–Zn–(Ni) particle (*a*) and XRF images of Ca, Ti, Fe and As (*b*). The number of inclusions clearly varies throughout the diamond. Scanned area: 7 µm × 7 µm; beam dimensions: 46 nm (V) × 50 nm (H); step size: 40 nm; measurement time: 1 s per point [Reprinted with permission from Laforce *et al.* (2014 ▸): Copyright 2014, American Chemical Society.]

As shown in Fig. 9(*a*) ▸, as well as Ca, Ti, Fe and As, several diamond inclusions present Cu, Zn and, to a lesser extent, Ni, which are elements not typical of deep Earth material. Data analysis by K-means clustering and PCA routines (Laforce *et al.*, 2014 ▸) indicated that these elements are present as large individual particles (0.5 µm^2^), suggesting the formation of a secondary phase, not originating from the lower mantle but from a later stage during its ascent to the surface.

As displayed in Fig. 9(*b*) ▸, most inclusions are located in the bottom left corner of the images, with the concentration of inclusions varying throughout the diamond. The Fe map reveals the presence of iron-rich inclusions and an intense iron signal toward the upper right corner of the image. This finding is crucial to the hypothesis of the presence of ringwoodite in this diamond.

### 3.3. Spatially resolved composition of Cu(In,Ga)Se_2_ thin-film solar cells   

**Figure 10:**
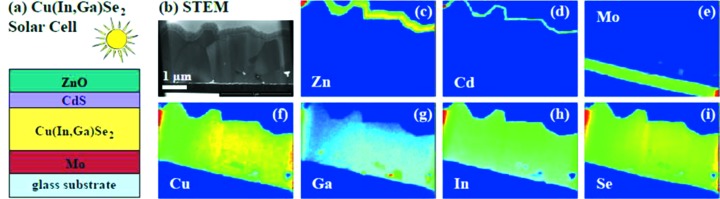
(*a*) Schematic of the structure of a CIGS solar cell. (*b*) Element maps of a complete CIGS cross section studied by nano-XRF imaging. [Reprinted with permission from Schöppe *et al.* (2015 ▸): Copyright 2015, American Institute iof Physics.]

Among the materials used for thin-film solar cells, Cu(In,Ga)Se_2_ (CIGS) reached the highest conversion efficiencies (20% in the laboratory) in 2014 (Chirilă *et al.*, 2011 ▸; Green *et al.*, 2015 ▸). A typical CIGS thin-film solar cell consists of a Mo back contact, a CIGS absorber layer, a CdS buffer layer, and a transparent ZnO front contact, as shown in Fig. 10(*a*) ▸. Due to their polycrystalline nature with typical grain sizes of 0.5–1 µm, the CIGS absorber layers present lateral heterogeneity as well as a pronounced Ga gradient on a depth scale determined by the preparation process. Consequently several properties are affected such as the material band gap, the charge carrier mobility and recombination. Detailed knowledge of the chemical spatial variation is indispensable for a comprehensive understanding of their functionality.

XRF nanoimaging was performed at ID16B using a 50 nm beam with an incident energy of 17.5 keV. Thin (<1 µm) cross-section lamellas of CIGS produced by a sequential process (Oertel, 2012 ▸) were prepared using a dual focused ion beam system.

The results shown in Fig. 10(*b*) ▸ reveal the formation of a MoSe_2_ layer at the interface between the Mo contact and the CIGS layer, as well as gallium and copper variations at grain boundaries. The observed Se signal is not limited to the absorber layer, but is also clearly detected inside the Mo back contact, especially under the detached regions of the absorber layer. Such findings help to optimize the preparation conditions required to further increase the conversion efficiency and to fully exploit the potential of these thin film solar cells (Schöppe *et al.*, 2015 ▸). The approach of measuring thin lamellas prepared by focused ion beam using nano-XRF can also be applied to other multilayer structures or inhomogeneous materials such as kesterites.

## 4. Short-term activities   

The design of the hard X-ray nanoprobe ID16B will evolve within the coming months towards the implementation of *in situ* capabilities. Instrument developments are currently under development in order to perform ever more challenging research and to enlarge the experimental capabilities. These include the possibility of carrying out X-ray nano-analysis at energies above 33 keV, X-ray nanotomography under variable temperatures using a dedicated micro-heater, and simultaneous acquisition of XEOL and XRF at very low temperatures using a helium mini-cryostat (Schöppe *et al.*, 2015 ▸).

## 5. Conclusions   

A new state-of-the-art nanoprobe for hard X-ray nano-analysis and 2D/3D X-ray imaging at submicrometer length scales has been built and successfully commissioned at the ESRF. Covering a large variety of research areas, the ID16B beamline offers a multimodal approach, nanometer-scale spatial resolutions, offering X-ray excitation energies from 6.5 to 70 keV. Its design integrates high-performance focusing optics, reliable high-precision scanning stages, and efficient detection schemes all within a temperature stabilized environment. Further short-term developments include *in situ* capabilities, in particular, temperature-dependent sample environment conditions. The first scientific results obtained demonstrate the great potential of the new ID16B facility.

## Figures and Tables

**Table 1:** Main characteristics of the U26 in-vacuum undulator

Period (mm)	26
Length (m)	2.5
Magnet material	Sm_2_Co_17_
Minimum gap (mm)	6.5
Peak field at minimum gap (T)	0.935
Deflection parameter at minimum gap	2.27
Fundamental energy (keV)	3.73
Total power emitted at minimum gap (*I* = 0.2 A) (kW)	10.1
